# Administration of a Synbiotic Containing *Enterococcus faecium* Does Not Significantly Alter Fecal Microbiota Richness or Diversity in Dogs With and Without Food-Responsive Chronic Enteropathy

**DOI:** 10.3389/fvets.2019.00277

**Published:** 2019-08-30

**Authors:** Rachel Pilla, Blake C. Guard, Joerg M. Steiner, Frederic P. Gaschen, Erin Olson, Dirk Werling, Karin Allenspach, Silke Salavati Schmitz, Jan S. Suchodolski

**Affiliations:** ^1^Gastrointestinal Laboratory, Department of Small Animal Clinical Sciences, College of Veterinary Medicine and Biomedical Sciences, Texas A&M University, College Station, TX, United States; ^2^Department of Veterinary Clinical Sciences, School of Veterinary Medicine, Louisiana State University, Baton Rouge, LA, United States; ^3^Department of Pathobiology and Population Sciences, Royal Veterinary College, University of London, North Mymms, United Kingdom; ^4^Department of Veterinary Clinical Sciences, College of Veterinary Medicine, Iowa State University, Ames, IA, United States; ^5^Royal (Dick) School of Veterinary Studies & The Roslin Institute, College of Medicine and Veterinary Medicine, University of Edinburgh, Midlothian, United Kingdom

**Keywords:** *Enterococcus*, diarrhea, inflammatory bowel disease, probiotics, microbiome

## Abstract

**Background:** Canine chronic enteropathies (CE) are a group of intestinal diseases that can be categorized based on treatment response into diet- or food- responsive enteropathy (FRD), antibiotic-responsive enteropathy, steroid-responsive enteropathy, and non-responsive enteropathy. CE can often be associated with intestinal dysbiosis and thus administration of probiotic or synbiotic products may provide a useful tool for the management of some of these patients. *Enterococcus faecium* (EF) is a probiotic strain included in a commercially available synbiotic for small animals, however its impact on the microbial communities in dogs with FRD has not yet been evaluated.

**Hypothesis/Objectives:** The administration of a synbiotic will lead to a significant difference of the fecal microbial composition and/or diversity in dogs with FRD, and these changes are not attributable to diet change alone.

**Animals/Samples:** Twelve dogs with FRD fed a hydrolyzed protein diet received either a synbiotic (EF, fructooligosaccharides, gum Arabic) or placebo. Fecal samples were taken before and 6 weeks into treatment. Fecal samples were also acquired from 8 healthy dogs before and 6 weeks after being switched to the same hydrolyzed protein diet as their sole food.

**Methods:** Bacterial DNA was extracted from fecal samples and next generation sequencing based on the 16S rRNA genes was performed. Microbial composition and diversity between groups were compared using QIIME.

**Results:** There was a small increase in species diversity in the feces of dogs with FRD treated with synbiotics. However, there were no significant differences in microbial community composition before and after 6 weeks in either the synbiotic or placebo treated dogs with FRD or the healthy dogs. In all groups, large individual variations were observed.

**Conclusions:** No changes in microbial composition were observed in diseased or healthy dogs with diet change alone. However, administration of a synbiotic increased bacterial richness in both groups.

## Introduction

Canine chronic enteropathies (CE) are a group of intestinal diseases of unknown cause (1, 2). They are usually classified by response to treatment as food-responsive disease (FRD), antibiotic-responsive disease (ARD), and steroid-responsive disease (SRD), with the latter also being termed (idiopathic) inflammatory bowel disease (IBD) (2, 3). All of these syndromes manifest in variable degrees and combinations of gastrointestinal signs (i.e., diarrhea, vomiting, weight loss, changes in appetite).

All dogs with CE present with intestinal inflammation to some degree and have been shown to share similar alterations in the microbiome when compared to healthy dogs (4). Those alterations in the gut microbiome composition are termed dysbiosis, especially when they result in functional changes in the microbial transcriptome, proteome, or metabolome (5). It has been speculated that oxygen, which is increased in the GI tract of dogs with CE due to inflammation, might be responsible for the observed dysbiosis (6). This hypothesis focuses on the availability of oxygen in the intestinal lumen (5), which negatively impacts strict anaerobe populations, and drives an uncontrolled luminal expansion of facultative anaerobes, especially members of the *Enterobacteriaceae* family (5). The increase in abundance of facultative anaerobic bacteria belonging to the *Enterobacteriaceae* family is a common marker of dysbiosis in dogs (7).

In dogs with IBD treated with immunosuppressive therapy, with or without antibiotics or other therapeutic measures, clinical recovery is not always accompanied by a recovery of the microbial dysbiosis. In one study (8), although all dogs clinically recovered, the diversity indices for the fecal bacterial communities present after 3 weeks of therapy showed a trend toward a further decrease.

Although antibiotics like metronidazole or tylosin are prescribed to many dogs with chronic diarrhea, this practice is of increasing concern. The true incidence of ARD is reported to be low (8–16%) (9, 10), thus administering antibiotics empirically likely leads to their overuse, directly or indirectly contributing to global antimicrobial resistance. It is widely accepted that antibiotic administration in general causes changes in the composition and richness of the intestinal microbiota in people (11) and companion animals (12), and this dysbiosis can be detrimental for overall host health. Administration of oral tylosin to healthy dogs was associated with microbiota alterations that were still present 14 days after withdrawal (13), suggesting a possible long-term adverse effect in some animals. Fecal bacterial diversity was also reduced after oral administration of metronidazole to healthy dogs (14). Overall, the necessity of avoiding empirical and injudicious use of antibiotics in dogs with chronic diarrhea cannot be overemphasized.

To aid recovery of dysbiosis in dogs with CE and as alternative to antibiotic treatment (4–6), modification of the intestinal microbiota in the form of pre- and probiotics (combinations of both are called synbiotics) seems an interesting and desirable treatment option. Several studies have shown that probiotics can influence key biological signaling pathways of inflammation in immune cells in humans and rodent models (7, 15–18), as well as in dogs (19).

In one study, treatment of IBD in dogs with a probiotic induced remission comparable with combined therapy (immunosuppressive combined with antibiotic) (8). In addition, when combined with immunosuppressive therapy, probiotics were found to upregulate tight junction protein expression in dogs with IBD, suggesting a beneficial effect on mucosal homeostasis (19).

While dogs affected by FRD or IBD have similarly dysbiotic microbiomes, their response to treatment can be somewhat different. In one study, no significant changes in microbial communities were found after treatment between dogs with FRD treated with an elimination diet with a novel protein source and dogs with IBD treated with a combination of diet and immunosuppressant therapy (20). However, in another study (21), the diversity of the fecal microbiome increased after feeding a vegetable diet to dogs with FRD and after treatment the fecal microbiome was no longer different from healthy controls. The difference between these studies may be attributable to the sampling schedule: in the first study (20), dogs where sampled after 14 days, and in the latter (21), they were fed the vegetable diet for 6 weeks before sampling. We have recently published studies looking at the clinical effectiveness of *Enterococcus faecium* (EF) in dogs with FRD, as well as assessing the influence of this allegedly probiotic bacterium on several inflammatory pathways in canine CE (22, 23). EF strain NCIMB 10415 4b1707 is widely available as a commercial small animal probiotic or synbiotic in Europe and in the USA. So far, EF has not convincingly shown a clinical benefit over dietary change alone in FRD cases (23). However, the effect of EF on the composition and diversity of the fecal microbiome of dogs with CE has not yet been evaluated.

The hypothesis of the current study was that administration of an *E. faecium* containing synbiotic product will lead to a significant difference of the fecal microbial composition and/or diversity in dogs with FRD, and these changes are not attributable to diet change alone.

## Materials and Methods

### Animal Enrolment and Sample Collection

#### Dogs With Food-Responsive Chronic Enteropathy

The synbiotic clinical trial in dogs with FRD was conducted between June 2010 and May 2013. Details of inclusion/exclusion criteria and diagnostic workup as well as partial results of this trial have been partially published elsewhere (22). The synbiotic used (Synbiotic D-C; Probiotics International Ltd. [Protexin], Somerset, UK) contained 1 × 10^9^cfu EF strain NCIMB 10415 4b1707 per capsule, plus the prebiotics fructooligosaccharides (FOS) and gum Arabic. The placebo consisted of an identical capsule containing maltodextrin. A commercially available hydrolyzed protein diet (Purina Veterinary Diet canine HA Hypo Allergenic, Nestle/Purina, York, UK) was used as elimination diet throughout the study period.

Spontaneously voided fecal samples from these dogs with FRD were collected at the initial visit and approximately 6 weeks after starting the diet and trial medication (synbiotic or placebo), to which the dogs were randomized before the start of the study in a double-blinded fashion.

#### Healthy Control Dogs

To further ensure that microbiota changes anticipated upon additional synbiotic treatment in dogs with FRD were not due to the diet change, a group of healthy control dogs were recruited to assess the effect of diet change alone as part of an unrelated study at LSU. This study was conducted between May and September 2014. All dogs were student and staff-owned and deemed healthy based on physical examination and a minimal database, consisting of a complete blood count, serum chemistry profile, and urine specific gravity. Exclusion criteria included a history of digestive disease in the 12 months before recruitment. All of those dogs were given a broad spectrum anthelminthic (fenbendazole, 50 mg/kg PO once daily) for 3 days prior to the study.

Fecal samples from these healthy dogs (*n* = 8) were collected before and after 6 weeks of receiving the same hydrolyzed protein diet as their sole food (all dogs were on different commercially available dog foods before).

#### Fecal Sample Collection

All freshly voided fecal samples were frozen at −80° C immediately after collection and shipped to the Gastrointestinal Laboratory at Texas A&M University on dry ice as a batch.

### Microbiota Analysis

Bacterial DNA extraction from fecal samples was performed using a commercially available kit (Power Soil® DNA isolation kit, Mo Bio Laboratories Inc., Quiagen, Carlsbad, CA, USA) according to the manufacturer's instructions and as described elsewhere (18). Bacterial tag-encoded FLX-titanium amplicon pyrosequencing (bTEFAP) based on the V1–V3 region (*E. coli* position 27–519) of the 16 S rRNA gene was performed on samples from dogs with FRD as described previously, with forward primer 28F: GAGTTTGATCNTGGCTCAG and reverse primer 519R: GTNTTACNGCGGCKGCTG (5, 24). Raw sequence data were screened, trimmed, denoised, filtered, and chimera depleted with the QIIME (Quantitative Insights Into Microbial Ecology, www.qiime.org) pipeline version 1.7 with similar settings as published previously (5, 24). Operational taxonomic units (OTUs) were defined as sequences with at least 97% similarity using QIIME.

Healthy control dogs receiving the hydrolyzed protein diet alone were part of a subset of an unrelated study and conducted at a later time. For these samples, Illumina sequencing was performed with the following primers: 515F (GTGCCAGCMGCCGCGGTAA) and 806R (GGACTACVSGGGTATCTAAT). Raw sequence data was similarly screened, trimmed, denoised, filtered, and chimera depleted with the QIIME pipeline version 1.9, similar to the pipeline described above for dogs with FRD.

Analysis of sequencing data was done for all samples before and after 6 weeks of dietary and/or synbiotic intervention. A combined analysis with all 3 subgroups of dogs (FRD on diet and synbiotic, FRD on diet and placebo, healthy dogs with diet change) was not performed due to the different sequencing primers used.

Alpha (α) diversity was assessed as a measure of species richness in all samples. The chao1, Shannon index and observed species data were generated as described previously (4, 6) to generate the α-rarefaction plots and data. The OTU table was rarefied at 8,403 sequences/sample for samples of dogs with FDR, and 19,000 sequences for sample for the HC samples. Phylogeny-based UniFrac distance metric analysis was used to investigate differences in microbial communities as a measure of beta (β)- diversity. For this, the analysis of similarity (ANOSIM) function in a statistical software package (PRIMER 6, PRIMER-E Ltd., Luton, UK) was used on the UniFrac distance matrixes, both weighted and unweighted. Individual bacterial abundances were assessed first for normality using the Shapiro-Wilk Test and found to be non-parametric. Therefore, a Wilcoxon rank-sum test was used for taxonomic analysis of paired samples. *p*-values were adjusted for multiple comparisons by the Benjamin & Hochberg FDR. Statistical significance was set at *p* < 0.05.

Linear discriminant analysis effect size (LEfSe) was used to elucidate bacterial taxa that were associated with each diet trial. LEfSe was calculated using Calypso, a web-based software package that allows mining and visualizing of microbiome-host interactions (25).

## Results

### Animals

A total of 12 dogs of various breeds diagnosed with chronic enteropathy completed the randomized placebo-controlled treatment trial as part of another study and received a full standard workup (22). These dogs were newly diagnosed and all received the same hydrolyzed complete dog food. They were only included in the trial if they showed a full clinical response, and hence were classified as food-responsive. Dog breeds included Labrador Retrievers (*n* = 6), Golden Retrievers (*n* = 2), and one dog each of the following breeds: Bracco Italiano, English Setter, Miniature Schnauzer, and Standard Poodle. Six dogs were intact males, 2 dogs were castrated males, and 4 dogs were spayed females. The median age was 40 months (range 12–84 months). Seven dogs had been randomly assigned to receive the synbiotic product and 5 to receive a placebo.

In addition, a total of 8 healthy dogs were recruited that underwent dietary change to the same diet as the diseased populations, which included mixed breeds (*n* = 3), Mastiff (*n* = 2) and one each of the following breeds: Bull Terrier, Dachshund, and Hungarian Vizsla. Four dogs were castrated males, 2 intact females and 2 spayed females. The median age of the healthy controls was 42 months (range 24–72 months).

### Microbiota Analysis

**Figure 1:**
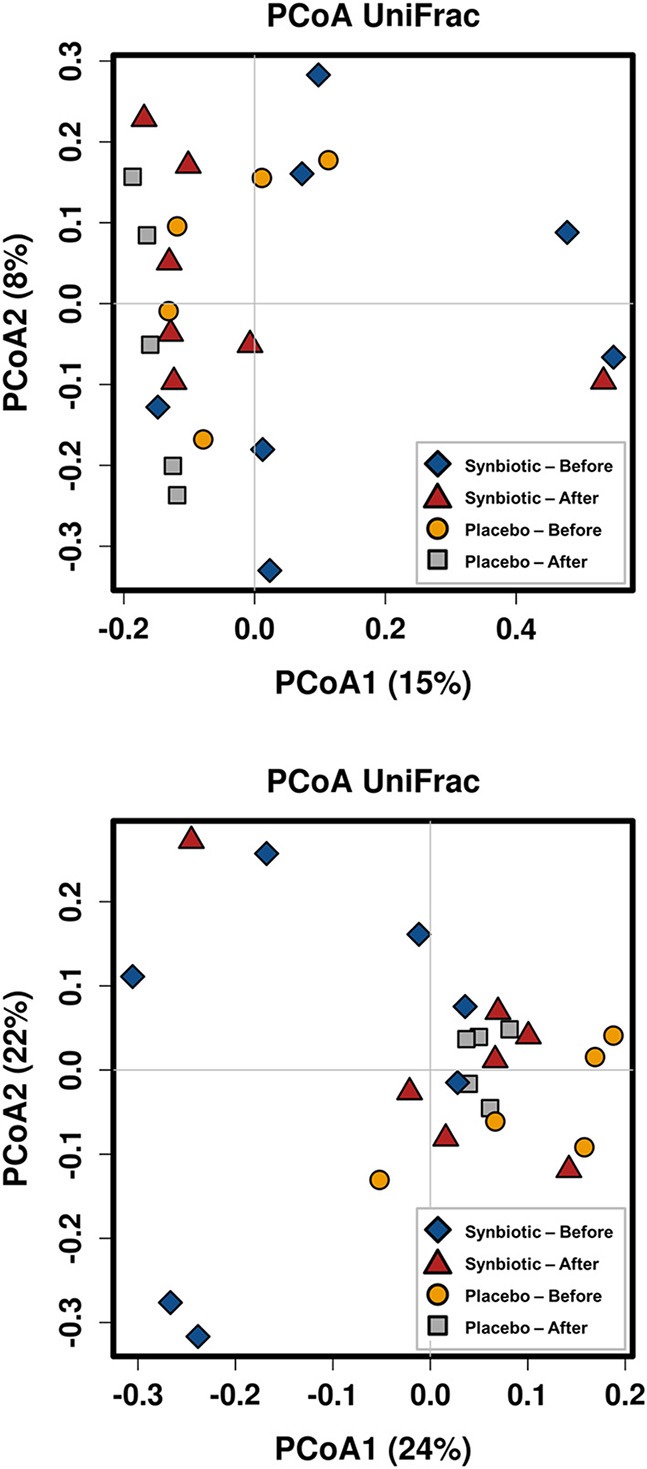
Principal coordinate analysis of the fecal microbiota from dogs with food-responsive chronic enteropathy. Beta diversity was calculated based on unweighted UniFrac distances (**A**, *p* = 0.205) and weighted UniFrac distances (**B**, *p* = 0.107).

**Figure 2:**
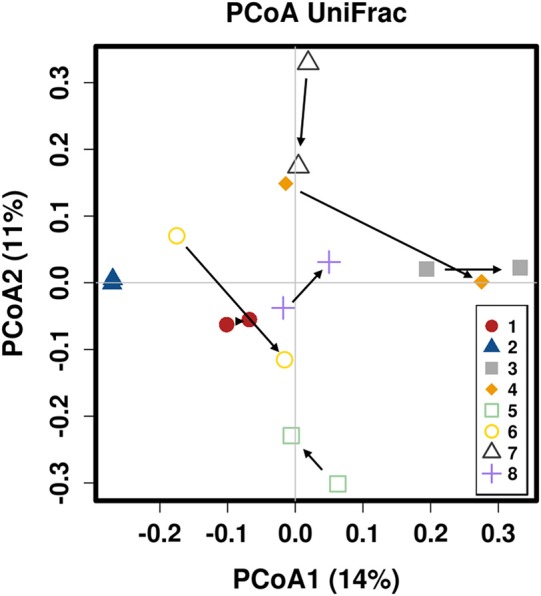
Principal coordinate analysis of the fecal microbiota from healthy dogs switched to hydrolyzed protein diet. Colors are marking individual dogs. Arrows indicate change from day 0 (before diet change) to 6 weeks after diet change.

**Figure 3:**
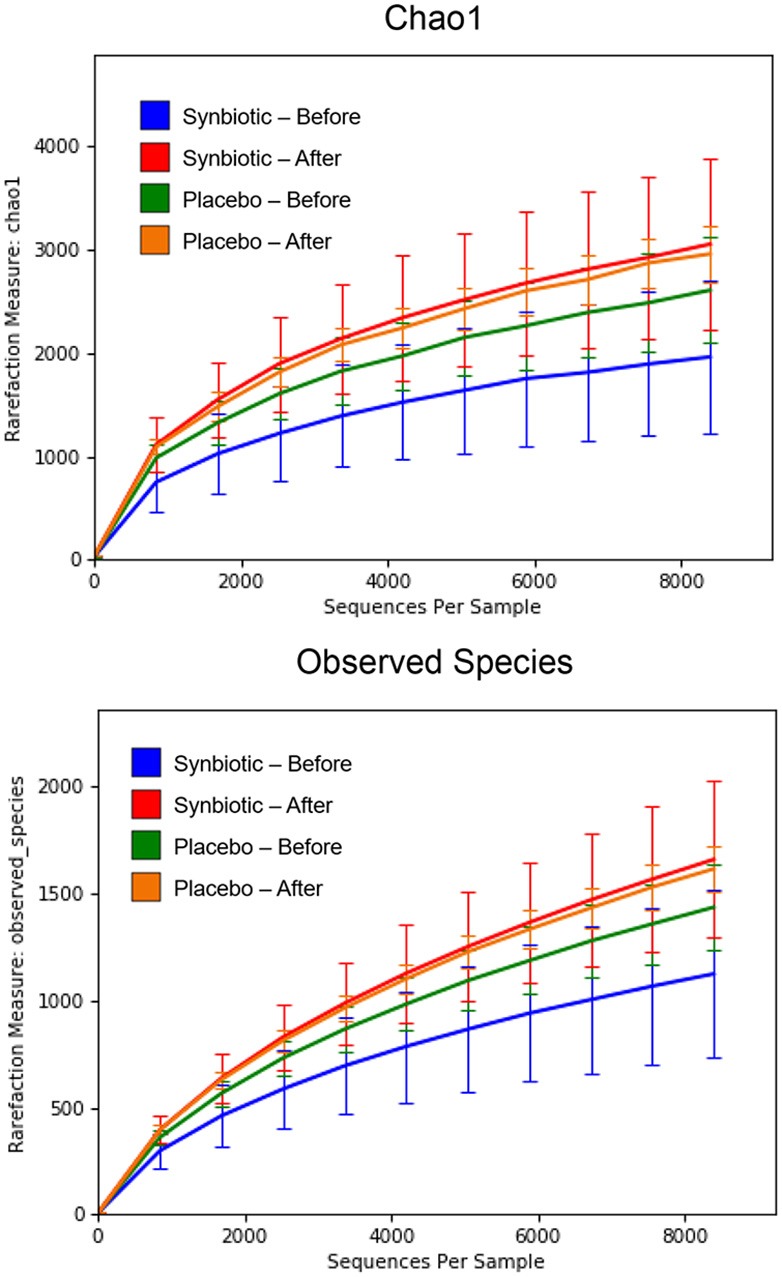
Rarefaction plot of fecal bacterial species from dogs with food-responsive chronic enteropathy. Data shown are from before and 6 weeks after a change to a hydrolyzed protein diet as a sole food, supplemented with either a synbiotic or a placebo, and expressed by the Chao 1 diversity index **(Upper)** and the number of observed bacterial species **(Lower)**.

**Figure 4:**
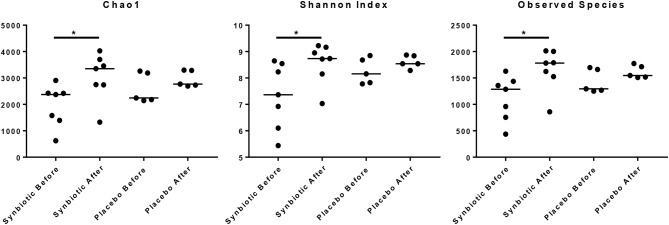
Species richness of fecal samples from dogs with food-responsive chronic enteropathy. Chao1, Shannon index and observed species before and after 6 weeks of treatment with hydrolyzed protein diet and synbiotics are shown.

**Figure 5:**
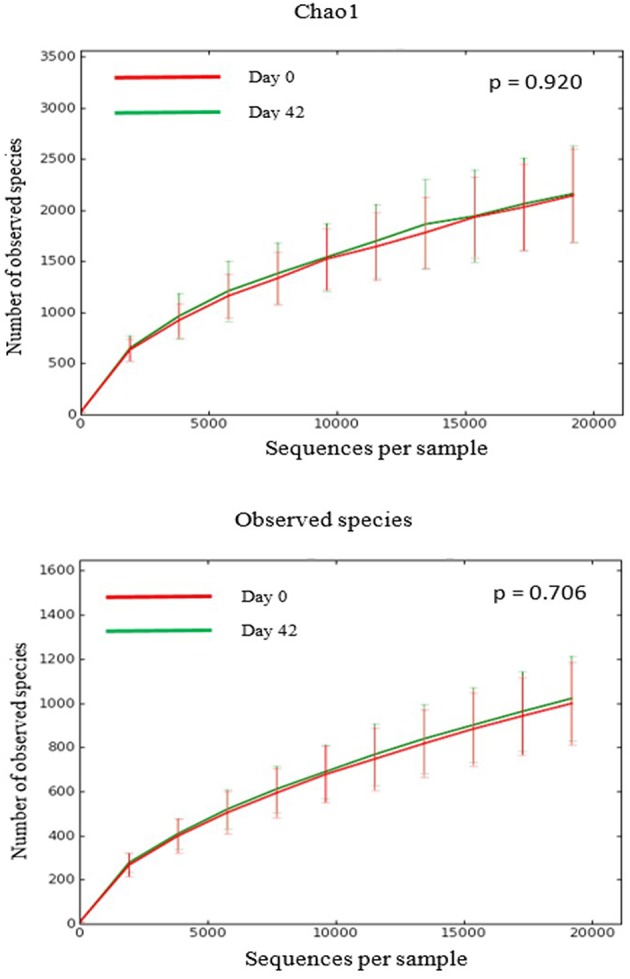
Rarefaction plots of fecal bacterial species from healthy dogs. Data shown are from before (red lines) and 6 weeks after (green lines) a change to a hydrolyzed protein diet as a sole food, and expressed by the Chao1 diversity index (upper panel) and the number of observed bacterial species (lower panel).

There was no difference in the overall microbiota taxonomic composition (β-diversity, unweighted UniFrac) before and after administration of the hydrolyzed protein diet with either placebo or synbiotic in dogs with FRD (ANOSIM unweighted *p* > 0.071, see Figure 1A). However, when microbiota taxonomic composition was evaluated together with taxa abundance (weighted UniFrac, see Figure 1B), there was a significant difference between the two groups at baseline (*p* = 0.038), and in the placebo group before and after treatment (*p* = 0.016). No difference was found on β-diversity for healthy controls fed the hydrolyzed protein diet for the same 6-weeks period (ANOSIM unweighted *p* = 0.836; weighted *p* = 0.260). However, it is important to note that there was a large inter-individual variation between subjects, which can be appreciated in Figure 2. While some dogs remained within the same quadrant after diet change, others showed a larger variation, as indicated by the length of the arrows. Bacterial richness (α-diversity) was found to be significantly increased in the group of dogs with FRD treated with diet and the synbiotic vs. the placebo-treated dogs (see Figures 3, 4). Rarefaction curves are shown in Figure 3 and indicate that the rarefaction depth was appropriate for the analysis. In Figure 4, α-diversity parameters Chao1, Shannon Index, and Observed Species are significantly increased in dogs with FRD treated with diet and synbiotic compared to baseline (*p* = 0.016, 0.031, and 0.016, respectively). Despite visible difference of baseline samples from the placebo and synbiotic groups in Figures 3, 4, no significance was seen (Chao1 *p* = 0.432 and Observed Species *p* = 0.343). Dogs from the healthy control group receiving the hydrolyzed protein diet alone did not show any variation on α-diversity parameters Chao1, Shannon Index, and Observed Species (see Figures 4, 5, *p* = 0.920, 0.165, and 0.706, respectively).

Linear discriminant analysis effect size (LEfSe) found an increase in the genus *Enterococcus* (family *Enterococcaceae*) to be associated with samples from dogs with FDR receiving the combination of the hydrolyzed protein diet and the synbiotic (see Table 1). Samples of dogs with FRD receiving treatment with hydrolyzed protein diet combined with placebo instead were found to be associated with an increase in the order *Clostridia*.

**Table 1:** Linear Discriminant Analysis (LDA) of bacterial taxa from canine fecal samples and their associations with synbiotic treatment.

**Bacterial Taxa**	**LDA Score**
**BEFORE SYNBIOTIC**	
Order *Lactobacillales*	5.01
**AFTER SYNBIOTIC**	
Family *Enterococcaceae*	4.20
Genus *Enterococcus*	3.88
**BEFORE PLACEBO**	
Class *Flavobacteriia*	4.84
Order *Flavobacteriales*	3.92
Family *Flavobacteriaceae*	4.03
Family *Bacillaceae*	3.52
Genus *Geobacillus*	3.61
Phylum *Fusobacteria*	5.24
Class *Fusobacteria*	4.84
Order *Fusobacteriales*	4.84
Family *Fusobacteriaceae*	4.80
Family *Fusobacteriaceae*, unidentified genus	4.71
Genus *Fusobacterium*	3.27
Family *Fusobacteriaceae*, genus J2-29	3.71
**AFTER PLACEBO**	
Phylum *Firmicutes*	5.12
Class *Clostridia*	5.25
Order *Clostridiales*	5.23

*Only LDA scores >3.5 are shown*.

Taxonomy analysis revealed no differences before and after treatment in dogs with FRD receiving diet and a synbiotic (see Supplementary Table 1). However, there was a significant decrease in *Actinobacteria* and *Bacteroidetes* in the group of dogs with FRD that did not receive the synbiotic treatment (corrected *p*-value 0.031 for both phyla) after 6 weeks (see Supplementary Table 2). In the group of healthy dogs receiving the hydrolyzed protein diet alone, diet change had no significant effect on distribution of bacterial taxa (see Supplementary Table 3).

## Discussion

The objective of the current study was to assess changes in fecal microbiota upon administration of a synbiotic combined with hydrolyzed protein diet in dogs with FRD compared to effects of diet change alone. Interestingly, even though the microbiota composition (β-diversity) was not significantly altered, bacterial richness as indicated by species diversity (α-diversity) increased with synbiotic treatment. This effect was not seen after switching either FRD or healthy dogs to the hydrolyzed protein diet alone.

When associations were investigated with LEfSe, the only bacterial taxa associated with treatment with the synbiotic was genus *Enterococcus*. This finding is not unexpected, as the study design did not include a washout period between the end of the synbiotic administration and the collection of samples. Samples from dogs receiving treatment with hydrolyzed protein diet and placebo, instead, were associated with an increase in the order *Clostridiales*. Many members of this order are known to be depleted in dogs with FRD and those with other chronic enteropathies (e.g., family *Lachnospiraceae*), and their increase may simply reflect a recovery from dysbiosis as described elsewhere (21).

In dogs with FRD that did not receive the synbiotic treatment there was a significant decrease in *Actinobacteria* and *Bacteroidetes* after 6 weeks of treatment with the hydrolyzed protein diet. The difference in those two phyla, however, is likely of no clinical significance, as it is not mirrored in changes on the levels of bacterial order, family, or genus. In the group receiving synbiotic treatment combined with the hydrolyzed protein diet, no significant changes were observed in any taxonomic level.

The lack of an effect of diet alone on the fecal microbiota diversity and composition in the healthy dogs is not unexpected. Even though other dietary factors have been shown to influence microbiota in rodent models and people (26, 27), these might need to be drastic (e.g., very high-fat or high protein diets). In a study with a diet that included exclusively vegetable proteins but maintained macronutrient composition within typical “maintenance” complete dry dog food, no significant changes were seen in abundance, or diversity of microbial communities of healthy dogs (21). Those results are in agreement with ours; and indicate that the protein source is not necessarily an important factor for gut microbial communities of healthy dogs. However, the same study (21) found that dogs with FRD had a significant increase in alpha-diversity, and microbial communities significantly changed both in composition and abundance when fed the vegetable-protein diet. Such alterations likely reflect the recovery of the microbiota once the irritating antigen is removed, rather than an effect of the diet itself.

Significant increases in fiber content have been shown to alter the canine fecal microbiota in a previous study (28). The hydrolyzed protein diet used in our study, however, had a low fiber content, which was not different from a standard “maintenance” complete dry dog food (around 2–4% dry matter), and the amount of FOS included in the synbiotic was not enough to significantly alter those percentages.

It is important to note that overall, amongst both healthy and diseased dogs, there was a large individual variation observed in microbiome parameters between the two time-points examined, similar to reports for people. In addition, despite randomized group assignation, dogs assigned to placebo or synbiotic groups significantly clustered separately at baseline, which further complicates the interpretation of our data (see Figure 1). It is well-known that large inter- and intra-individual variations in the intestinal and fecal microbiota occur (4, 5, 24, 27), which is also a possible explanation for the lack of overall effect on microbiota composition observed in this study. Hence it is possible that a larger number of dogs needs to be studied to “tease out” subtle changes of the microbiota that could be caused by administration of synbiotics. However, changes observed were accompanied by clinical recovery in both placebo and synbiotic groups and might still be meaningful to the individual host organism. However, there is currently no accepted way to assess those types of effects, which fall into the realm of personalized medicine, and it was not the aim of the study presented here to assess such effects.

In general, it is accepted that microbial diversity decreases in the chronically inflamed gut in both people and dogs (29–31). Hence, being able to increase richness might be one of the small measures that can be taken to counteract “dysbiosis” and inflammation. However, it is currently unclear which specific changes of microbial composition might improve clinical outcome in dogs with CE, and the increase in microbial species richness seen in the synbiotic-treated group can be of questionable impact.

With regards to the synbiotic used in this study, it is possible that more “aggressive” probiotic or synbiotic interventions (i.e., more frequent administration, higher dosages, different or multiple strains of probiotics, different types, and dosages of prebiotics) are necessary to increase the magnitude of the effect seen. Studies that demonstrated the benefit of probiotics alone (8) or in combination with standard therapy (19) in dogs with IBD administered at a dosage of 112–225 billion bacteria, including 7 different strains, for each 10 Kg of body weight (8), or 450 billion of the same bacteria mixture for 10–20 Kg of body weight. In comparison, our study included only one strain of EF, at the dosage of 1 billion bacteria per dog once daily, as recommended by the manufacturer, and in compliance with the European Foods Standard Agency (EFSA) regulation (EC) no 1,831/2,003, which regulates the use of probiotic bacteria in animal species, including dogs, and cats.

In addition, reversal of both microbial alterations and the associated inflammatory abnormalities seems to be a slow process, especially if influencing the microbiome by synbiotics is the only measure and no additional drugs with strong anti-inflammatory effects are used (8). It has been shown that despite a decrease in clinical activity indices, there were no changes in the fecal microbiota and serum metabolome after at least 3 weeks of treatment in dogs with IBD (31). This also fits with the observation, that the EF strain used in the synbiotic preparation did not lead to a difference in clinical improvement or anti-inflammatory gene expression profile in the intestinal mucosa of the same dog cohort within 6 weeks (22). It might therefore be necessary to observe and reassess these dogs after longer periods of treatment, where indicators of improvement of dysbiosis and clinical signs are more readily noted.

While next generation sequencing is an excellent tool to assess changes in bacterial phyla and genera in different populations of dogs, one limitation in the current study is that samples from the 2 study groups were analyzed in different runs and with slightly different techniques, which might have introduced variation. Also, Illumina sequencing might not be the ideal tool to look for more subtle changes of bacterial composition beyond phyla and genera. Species of bacteria present in low abundances that are either likely important for mucosal health [i.e., *Lactobacillus, Bifidobacterium, Faecalibacterium prausnitzii* (5, 32)] or have a potential pathogenic role (i.e., *E.coli, Clostridium perfringens*) are very difficult to assess without also employing more sensitive methods, for example qPCR (33).

Even though no significant changes in microbial composition were observed in diseased or healthy dogs with diet change alone, administration of a synbiotic increased bacterial richness in both groups. The EF strain's transient colonization might hence have significantly affected mucosal homeostasis as reported elsewhere (19). In addition, interactions between different bacterial taxa and even metabolites produced by them can activate metabolic pathways both in the host and in other bacterial species. Therefore, metabolomics approaches to chronic intestinal diseases assessing for example short chain fatty acids or an untargeted detection of metabolites might be more informative and a good target for future studies as exemplified by studies in humans (28).

## Conclusion

Overall, the results of this study show a small increase in species diversity in the feces of dogs with FRD treated with synbiotics. While we cannot quantify how much of this effect was a by-product of the administration of the probiotic itself, even small changes in composition may significantly impact metabolite production and, as consequence, intestinal health. Questions of which probiotic or synbiotic at what dose is most appropriate for the treatment of canine CE, and especially which parameters are most likely to represent therapeutic and clinical success remain open and the subject of further studies, however, pro- or synbiotics should be considered when empirical treatment trials are performed, especially as a substitute for potentially detrimental antibiotic trials.

## Data Availability

Microbial sequences generated and analyzed during the current study are available in the Sequence Read Archive (https://www.ncbi.nlm.nih.gov/sra) as SRP066795. The dataset regarding individual patient and control dog details are not publicly available due to data protection regulations, but are available from the corresponding author on reasonable request.

## Author Contributions

SS conducted the primary research by recruiting and acquiring samples from dogs with chronic enteropathy, which was part of a wider study assessing probiotic effects on this condition. She has also analyzed and interpreted the patient data and wrote the first draft of the manuscript. This study was planned and supported by KA and DW. FG and EO planned, recruited and acquired data and samples from healthy control dogs. BG and RP conducted the practical work involved in DNA extraction, sequencing and analysis of microbiota sequencing data under the supervision and guidance of JSt and JSu. Major contributions to the manuscript were from by RP, BG, and SS, with comments and corrections provided by all other authors (JSt, FG, EO, DW, KA, JSu).

### Conflict of Interest Statement

The authors declare that this study was part of a Ph.D. funded by a BBSRC studentship with Protexin Ltd. Somerset, UK. In addition, the authors declare that Nestlé-Purina Pet Care USA provided the diet used for the healthy control dogs at LSU (FG, EO). The funders had no role in study design, data collection and analysis, preparation of the manuscript, or decision to publish (even though they reserved the right to view any manuscript deriving from the Ph.D. work before publication). Parts of the probiotic clinical trial in dogs with FRE have already been published elsewhere (https://onlinelibrary.wiley.com/doi/full/10.1111/jvim.12563). The remaining authors declare that the research was conducted in the absence of any commercial or financial relationships that could be construed as a potential conflict of interest.
