# Supplementary material for: Administration of a Synbiotic Containing Enterococcus faecium Does Not Significantly Alter Fecal Microbiota Richness or Diversity in Dogs With and Without Food-Responsive Chronic Enteropathy
Source: Front Vet Sci. 2019 Aug 30;6:277. doi: 10.3389/fvets.2019.00277 (PMC6735529; doi:10.3389/fvets.2019.00277)
Supplement: Supplementary file 1 [file Table_1.DOCX]

| **Taxa diet change & synbiotics** | **Day 0** | | | **Day 42** | | | **p-value*** |
| --- | --- | --- | --- | --- | --- | --- | --- |
|  | **Min** | **Max** | **Median** | **Min** | **Max** | **Median** |  |
| **Phylum** |  | | | | | | |
| Actinobacteria | 0.00 | 64.84 | 0.88 | 0.00 | 2.72 | 0.19 | 0.305 |
| Bacteroidetes | 0.00 | 10.48 | 0.05 | 0.00 | 13.93 | 2.21 | 0.383 |
| Firmicutes | 34.91 | 99.49 | 62.92 | 76.20 | 95.72 | 94.26 | 0.383 |
| Fusobacteria | 0.00 | 0.29 | 0.02 | 0.00 | 5.30 | 0.58 | 0.383 |
| Proteobacteria | 0.07 | 42.18 | 2.24 | 0.27 | 23.71 | 1.39 | 0.701 |
| **Class** |  |  |  |  |  |  |  |
| Actinobacteria | 0.00 | 64.38 | 0.29 | 0.00 | 2.41 | 0.00 | 0.221 |
| Coriobacteriia | 0.00 | 7.12 | 0.24 | 0.00 | 1.60 | 0.19 | 0.221 |
| Bacteroidia | 0.00 | 10.48 | 0.05 | 0.00 | 13.93 | 2.21 | 0.221 |
| Bacilli | 0.80 | 33.67 | 8.05 | 0.24 | 15.32 | 1.65 | 0.348 |
| Clostridia | 11.86 | 89.11 | 49.67 | 60.88 | 94.89 | 80.77 | 0.362 |
| Erysipelotrichi | 0.00 | 17.38 | 4.96 | 0.00 | 11.16 | 1.36 | 0.672 |
| Fusobacteriia | 0.00 | 0.29 | 0.02 | 0.00 | 5.30 | 0.58 | 0.672 |
| Betaproteobacteria | 0.00 | 2.97 | 0.36 | 0.07 | 1.80 | 0.15 | 1.000 |
| Gammaproteobacteria | 0.02 | 39.22 | 0.85 | 0.02 | 21.88 | 0.71 | 1.000 |
| **Order** |  | | | | | | |
| Actinomycetales | 0.00 | 0.88 | 0.02 | 0.00 | 0.12 | 0.00 | 0.386 |
| Bifidobacteriales | 0.00 | 64.36 | 0.02 | 0.00 | 2.29 | 0.00 | 0.386 |
| Coriobacteriales | 0.00 | 7.12 | 0.24 | 0.00 | 1.60 | 0.19 | 0.386 |
| Bacteroidales | 0.00 | 10.48 | 0.05 | 0.00 | 13.93 | 2.21 | 0.503 |
| Bacillales | 0.00 | 1.05 | 0.02 | 0.00 | 0.19 | 0.00 | 0.539 |
| Gemellales | 0.00 | 0.83 | 0.02 | 0.00 | 0.02 | 0.00 | 0.539 |
| Lactobacillales | 0.17 | 33.65 | 7.88 | 0.10 | 15.27 | 1.02 | 0.539 |
| Turicibacterales | 0.00 | 4.77 | 0.02 | 0.00 | 0.63 | 0.10 | 0.539 |
| Clostridiales | 11.86 | 89.11 | 49.67 | 60.88 | 94.89 | 80.77 | 0.755 |
| Erysipelotrichales | 0.00 | 17.38 | 4.96 | 0.00 | 11.16 | 1.36 | 0.822 |
| Fusobacteriales | 0.00 | 0.29 | 0.02 | 0.00 | 5.30 | 0.58 | 1.000 |
| Burkholderiales | 0.00 | 2.97 | 0.36 | 0.07 | 1.80 | 0.15 | 1.000 |
| Enterobacteriales | 0.00 | 39.22 | 0.27 | 0.00 | 21.88 | 0.68 | 1.000 |
| **Family** |  | | | | | | |
| Bifidobacteriaceae | 0.00 | 64.36 | 0.02 | 0.00 | 2.29 | 0.00 | 0.577 |
| Coriobacteriaceae | 0.00 | 7.12 | 0.24 | 0.00 | 1.60 | 0.19 | 0.577 |
| Bacteroidaceae | 0.00 | 7.85 | 0.02 | 0.00 | 7.15 | 2.12 | 0.577 |
| Gemellaceae | 0.00 | 0.83 | 0.02 | 0.00 | 0.02 | 0.00 | 0.577 |
| Enterococcaceae | 0.00 | 19.21 | 0.05 | 0.05 | 13.06 | 1.02 | 0.577 |
| Lactobacillaceae | 0.00 | 4.40 | 0.24 | 0.00 | 0.34 | 0.05 | 0.577 |
| Streptococcaceae | 0.00 | 20.33 | 1.41 | 0.00 | 2.16 | 0.05 | 0.577 |
| Turicibacteraceae | 0.00 | 4.77 | 0.02 | 0.00 | 0.63 | 0.10 | 0.577 |
| Clostridiales; f_unclassified | 0.00 | 11.09 | 1.65 | 1.58 | 11.65 | 5.35 | 0.577 |
| Clostridiaceae | 0.27 | 26.48 | 8.05 | 2.38 | 51.18 | 6.93 | 0.597 |
| Lachnospiraceae | 0.07 | 74.74 | 24.22 | 0.44 | 78.29 | 61.17 | 0.663 |
| Peptostreptococcaceae | 0.07 | 46.78 | 0.24 | 0.00 | 7.66 | 1.00 | 1.000 |
| Ruminococcaceae | 0.00 | 2.99 | 0.44 | 0.02 | 13.81 | 1.99 | 1.000 |
| Erysipelotrichaceae | 0.00 | 17.38 | 4.96 | 0.00 | 11.16 | 1.36 | 1.000 |
| Fusobacteriaceae | 0.00 | 0.29 | 0.02 | 0.00 | 5.30 | 0.58 | 1.000 |
| Burkholderiales; f_unclassified | 0.00 | 2.53 | 0.02 | 0.00 | 1.24 | 0.07 | 1.000 |
| Alcaligenaceae | 0.00 | 0.44 | 0.02 | 0.00 | 0.56 | 0.15 | 1.000 |
| Enterobacteriaceae | 0.00 | 39.22 | 0.27 | 0.00 | 21.88 | 0.68 | 1.000 |
| **Genus** |  | | | | | | |
| Bifidobacterium | 0.00 | 64.09 | 0.02 | 0.00 | 2.02 | 0.00 | 0.651 |
| Collinsella | 0.00 | 6.59 | 0.15 | 0.00 | 1.36 | 0.10 | 0.651 |
| Bacteroides | 0.00 | 7.85 | 0.02 | 0.00 | 7.15 | 2.12 | 0.651 |
| Enterococcus | 0.00 | 18.60 | 0.05 | 0.05 | 12.69 | 0.97 | 0.651 |
| Lactobacillus | 0.00 | 4.40 | 0.24 | 0.00 | 0.34 | 0.05 | 0.651 |
| Streptococcus | 0.00 | 20.33 | 1.41 | 0.00 | 2.16 | 0.05 | 0.651 |
| Turicibacter | 0.00 | 4.77 | 0.02 | 0.00 | 0.63 | 0.10 | 0.651 |
| Clostridiales; f_unclassified; g_unclassified | 0.00 | 11.09 | 1.65 | 1.58 | 11.65 | 5.35 | 0.651 |
| Clostridiaceae; g_unclassified | 0.05 | 6.95 | 3.87 | 1.26 | 16.65 | 3.36 | 0.651 |
| Clostridium | 0.17 | 21.40 | 2.87 | 0.66 | 33.07 | 2.26 | 0.651 |
| SMB53 | 0.02 | 1.31 | 0.61 | 0.05 | 2.70 | 0.58 | 0.682 |
| Lachnospiraceae; g_unclassified | 0.00 | 43.93 | 6.47 | 0.07 | 56.48 | 29.22 | 0.718 |
| Blautia | 0.00 | 12.11 | 3.94 | 0.05 | 48.21 | 11.99 | 0.810 |
| Coprococcus | 0.00 | 7.03 | 1.63 | 0.02 | 1.73 | 0.58 | 0.872 |
| Dorea | 0.00 | 13.23 | 3.06 | 0.22 | 9.97 | 3.45 | 0.987 |
| Epulopiscium | 0.00 | 1.39 | 0.12 | 0.07 | 1.95 | 0.75 | 0.987 |
| [Ruminococcus] | 0.02 | 4.64 | 0.51 | 0.00 | 8.68 | 3.06 | 0.987 |
| Peptostreptococcaceae; g_unclassified | 0.07 | 46.61 | 0.22 | 0.00 | 7.05 | 0.88 | 0.987 |
| Clostridium | 0.00 | 0.10 | 0.02 | 0.00 | 0.58 | 0.10 | 0.987 |
| Ruminococcaceae; g_unclassified | 0.00 | 2.60 | 0.41 | 0.00 | 11.31 | 1.73 | 0.987 |
| Erysipelotrichaceae; g_unclassified | 0.00 | 0.71 | 0.05 | 0.00 | 1.19 | 0.00 | 0.987 |
| [Eubacterium] | 0.00 | 17.14 | 3.77 | 0.00 | 8.68 | 0.22 | 0.987 |
| Fusobacterium | 0.00 | 0.29 | 0.02 | 0.00 | 5.30 | 0.58 | 0.987 |
| Burkholderiales; f_unclassified; g_unclassified | 0.00 | 2.53 | 0.02 | 0.00 | 1.24 | 0.07 | 0.987 |
| Sutterella | 0.00 | 0.44 | 0.02 | 0.00 | 0.56 | 0.15 | 0.987 |
| Enterobacteriaceae; g_unclassified | 0.00 | 39.17 | 0.27 | 0.00 | 21.78 | 0.68 | 1.000 |

**Supplementary table 1.** Bacterial taxa detected in fecal samples from dogs with food-responsive chronic enteropathy, treated with hydrolyzed protein diet and synbiotic. * p-values have been adjusted for multiple comparisons by the Benjamin & Hochberg FDR
