# Supplementary material for: Administration of a Synbiotic Containing Enterococcus faecium Does Not Significantly Alter Fecal Microbiota Richness or Diversity in Dogs With and Without Food-Responsive Chronic Enteropathy
Source: Front Vet Sci. 2019 Aug 30;6:277. doi: 10.3389/fvets.2019.00277 (PMC6735529; doi:10.3389/fvets.2019.00277)
Supplement: Supplementary file 2 [file Table_2.DOCX]

| **Taxa diet change & placebo** | **Day 0** | | | **Day 42** | | | **p-value*** |
| --- | --- | --- | --- | --- | --- | --- | --- |
|  | **Min** | **Max** | **Median** | **Min** | **Max** | **Median** |  |
| **Phylum** |  | | | | | | |
| Actinobacteria | 0.00 | 1.00 | 0.44 | 0.07 | 0.32 | 0.29 | 0.031^a^ |
| Bacteroidetes | 0.32 | 17.72 | 7.37 | 0.05 | 1.82 | 1.29 | 0.031^a^ |
| Firmicutes | 51.79 | 95.65 | 79.09 | 96.72 | 99.66 | 98.08 | 0.118 |
| Fusobacteria | 1.82 | 10.82 | 7.71 | 0.12 | 0.78 | 0.44 | 0.118 |
| Proteobacteria | 0.07 | 30.90 | 2.72 | 0.00 | 1.02 | 0.15 | 0.143 |
| **Class** |  |  |  |  |  |  |  |
| Actinobacteria | 0.00 | 0.22 | 0.05 | 0.00 | 0.07 | 0.00 | 0.110 |
| Coriobacteriia | 0.00 | 0.95 | 0.44 | 0.07 | 0.32 | 0.27 | 0.165 |
| Bacteroidia | 0.32 | 17.72 | 7.34 | 0.05 | 1.82 | 1.29 | 0.213 |
| Bacilli | 0.02 | 21.47 | 1.63 | 0.27 | 3.82 | 0.80 | 0.213 |
| Clostridia | 29.91 | 86.92 | 71.99 | 82.49 | 95.33 | 87.24 | 0.250 |
| Erysipelotrichi | 0.19 | 20.47 | 1.02 | 1.19 | 16.90 | 7.03 | 0.315 |
| Fusobacteriia | 1.82 | 10.82 | 7.71 | 0.12 | 0.78 | 0.44 | 0.515 |
| Betaproteobacteria | 0.00 | 2.84 | 1.26 | 0.00 | 1.02 | 0.02 | 0.565 |
| Gammaproteobacteria | 0.07 | 29.56 | 0.68 | 0.00 | 0.12 | 0.00 | 0.676 |
| **Order** |  | | | | | | |
| Coriobacteriales | 0.00 | 0.95 | 0.44 | 0.07 | 0.32 | 0.27 | 0.069 |
| Bacteroidales | 0.32 | 17.72 | 7.34 | 0.05 | 1.82 | 1.29 | 0.069 |
| Bacillales | 0.02 | 0.12 | 0.07 | 0.00 | 0.02 | 0.00 | 0.135 |
| Lactobacillales | 0.00 | 21.35 | 0.29 | 0.00 | 0.10 | 0.02 | 0.164 |
| Turicibacterales | 0.00 | 1.56 | 0.10 | 0.17 | 3.79 | 0.78 | 0.164 |
| Clostridiales | 29.91 | 86.92 | 71.99 | 82.49 | 95.33 | 87.24 | 0.174 |
| Erysipelotrichales | 0.19 | 20.47 | 1.02 | 1.19 | 16.90 | 7.03 | 0.218 |
| Fusobacteriales | 1.82 | 10.82 | 7.71 | 0.12 | 0.78 | 0.44 | 0.289 |
| Burkholderiales | 0.00 | 2.82 | 1.22 | 0.00 | 1.02 | 0.02 | 0.360 |
| Enterobacteriales | 0.00 | 29.47 | 0.61 | 0.00 | 0.10 | 0.00 | 0.401 |
| Pseudomonadales | 0.00 | 0.10 | 0.05 | 0.00 | 0.00 | 0.00 | 0.401 |
| **Family** |  | | | | | | |
| Coriobacteriaceae | 0.00 | 0.95 | 0.44 | 0.07 | 0.32 | 0.27 | 0.116 |
| Bacteroidaceae | 0.10 | 17.43 | 7.17 | 0.05 | 1.51 | 1.22 | 0.116 |
| Porphyromonadaceae | 0.00 | 1.36 | 0.22 | 0.00 | 0.00 | 0.00 | 0.257 |
| Bacillaceae | 0.02 | 0.10 | 0.05 | 0.00 | 0.00 | 0.00 | 0.257 |
| Streptococcaceae | 0.00 | 2.16 | 0.17 | 0.00 | 0.10 | 0.00 | 0.257 |
| Turicibacteraceae | 0.00 | 1.56 | 0.10 | 0.17 | 3.79 | 0.78 | 0.257 |
| Clostridiales; f_unclassified | 2.41 | 12.81 | 6.61 | 5.74 | 9.48 | 7.22 | 0.257 |
| Clostridiaceae | 0.19 | 9.24 | 7.51 | 4.01 | 11.89 | 10.09 | 0.273 |
| Lachnospiraceae | 11.48 | 69.85 | 47.70 | 57.84 | 74.71 | 63.70 | 0.293 |
| Peptostreptococcaceae | 0.00 | 0.68 | 0.02 | 0.02 | 3.60 | 1.02 | 0.300 |
| Ruminococcaceae | 0.78 | 10.31 | 1.22 | 0.49 | 4.74 | 0.73 | 0.363 |
| Veillonellaceae | 0.00 | 10.87 | 0.41 | 0.00 | 0.17 | 0.10 | 0.375 |
| [Mogibacteriaceae] | 0.00 | 0.63 | 0.02 | 0.00 | 3.43 | 0.00 | 0.375 |
| Erysipelotrichaceae | 0.19 | 20.47 | 1.02 | 1.19 | 16.90 | 7.03 | 0.375 |
| Fusobacteriaceae | 1.82 | 10.82 | 7.71 | 0.12 | 0.78 | 0.44 | 0.375 |
| Burkholderiales; f_unclassified | 0.00 | 1.22 | 0.17 | 0.00 | 0.02 | 0.00 | 0.476 |
| Alcaligenaceae | 0.00 | 1.99 | 0.39 | 0.00 | 1.02 | 0.02 | 0.579 |
| Enterobacteriaceae | 0.00 | 29.47 | 0.61 | 0.00 | 0.10 | 0.00 | 0.778 |
| Moraxellaceae | 0.00 | 0.07 | 0.05 | 0.00 | 0.00 | 0.00 | 1.000 |
| **Genus** |  | | | | | | |
| Collinsella | 0.00 | 0.95 | 0.19 | 0.02 | 0.27 | 0.10 | 0.201 |
| Bacteroides | 0.10 | 17.43 | 7.17 | 0.05 | 1.51 | 1.22 | 0.201 |
| Parabacteroides | 0.00 | 1.36 | 0.22 | 0.00 | 0.00 | 0.00 | 0.205 |
| Bacillus | 0.00 | 0.05 | 0.02 | 0.00 | 0.00 | 0.00 | 0.205 |
| Geobacillus | 0.00 | 0.07 | 0.05 | 0.00 | 0.00 | 0.00 | 0.260 |
| Streptococcus | 0.00 | 2.16 | 0.17 | 0.00 | 0.10 | 0.00 | 0.260 |
| Turicibacter | 0.00 | 1.56 | 0.10 | 0.17 | 3.79 | 0.78 | 0.260 |
| Clostridiales; f_unclassified; g_unclassified | 2.41 | 12.81 | 6.61 | 5.74 | 9.48 | 7.22 | 0.260 |
| Clostridiaceae; g_unclassified | 0.00 | 6.15 | 4.04 | 0.63 | 7.25 | 5.69 | 0.260 |
| Clostridium | 0.19 | 4.16 | 1.77 | 2.63 | 4.45 | 3.06 | 0.260 |
| SMB53 | 0.00 | 1.02 | 0.24 | 0.07 | 1.63 | 0.68 | 0.260 |
| Lachnospiraceae; g_unclassified | 5.06 | 28.42 | 21.59 | 30.15 | 42.26 | 36.42 | 0.260 |
| Blautia | 2.36 | 19.28 | 13.62 | 11.55 | 19.30 | 12.96 | 0.347 |
| Coprococcus | 0.07 | 3.99 | 0.78 | 0.44 | 2.26 | 1.26 | 0.347 |
| Dorea | 2.48 | 21.98 | 9.73 | 3.79 | 15.75 | 5.01 | 0.347 |
| Epulopiscium | 0.02 | 0.95 | 0.22 | 0.41 | 2.24 | 1.63 | 0.515 |
| [Ruminococcus] | 1.02 | 4.89 | 2.84 | 1.12 | 4.98 | 3.11 | 0.515 |
| Peptostreptococcaceae; g_unclassified | 0.00 | 0.46 | 0.02 | 0.02 | 2.99 | 1.02 | 0.515 |
| Peptostreptococcus | 0.00 | 0.12 | 0.02 | 0.00 | 0.00 | 0.00 | 0.515 |
| Ruminococcaceae; g_unclassified | 0.58 | 8.10 | 1.09 | 0.36 | 3.99 | 0.71 | 0.634 |
| Butyricicoccus | 0.00 | 1.02 | 0.02 | 0.00 | 0.10 | 0.02 | 0.634 |
| Faecalibacterium | 0.00 | 1.07 | 0.02 | 0.00 | 0.56 | 0.02 | 0.730 |
| Oscillospira | 0.00 | 0.07 | 0.02 | 0.00 | 0.12 | 0.00 | 0.730 |
| Ruminococcus | 0.00 | 1.12 | 0.05 | 0.00 | 0.02 | 0.00 | 0.730 |
| Megamonas | 0.00 | 10.87 | 0.32 | 0.00 | 0.07 | 0.02 | 0.888 |
| [Mogibacteriaceae]; g_unclassified | 0.00 | 0.63 | 0.02 | 0.00 | 3.43 | 0.00 | 0.888 |
| Erysipelotrichaceae; g_unclassified | 0.00 | 1.17 | 0.15 | 0.05 | 6.71 | 0.24 | 0.888 |
| [Eubacterium] | 0.05 | 18.09 | 0.41 | 0.27 | 12.25 | 6.61 | 0.888 |
| Fusobacterium | 1.82 | 10.82 | 7.71 | 0.12 | 0.78 | 0.44 | 0.888 |
| Burkholderiales; f_unclassified; g_unclassified | 0.00 | 1.22 | 0.17 | 0.00 | 0.02 | 0.00 | 0.888 |
| Sutterella | 0.00 | 1.99 | 0.39 | 0.00 | 1.02 | 0.02 | 0.888 |
| Enterobacteriaceae; g_unclassified | 0.00 | 29.44 | 0.61 | 0.00 | 0.10 | 0.00 | 0.942 |
| Acinetobacter | 0.00 | 0.07 | 0.05 | 0.00 | 0.00 | 0.00 | 1.000 |

**Supplementary table 2.** Bacterial taxa detected in fecal samples from dogs with food-responsive chronic enteropathy, treated with hydrolyzed protein diet and placebo. * p-values have been adjusted for multiple comparisons by the Benjamin & Hochberg FDR. ^a^ = significant difference in taxa abundance between day 0 and day 42.
