# Supplementary material for: Administration of a Synbiotic Containing Enterococcus faecium Does Not Significantly Alter Fecal Microbiota Richness or Diversity in Dogs With and Without Food-Responsive Chronic Enteropathy
Source: Front Vet Sci. 2019 Aug 30;6:277. doi: 10.3389/fvets.2019.00277 (PMC6735529; doi:10.3389/fvets.2019.00277)
Supplement: Supplementary file 3 [file Table_3.DOCX]

| **Taxa healthy dogs** | **Day 0** | | | **Day 42** | | | **p-value*** |
| --- | --- | --- | --- | --- | --- | --- | --- |
|  | **Min** | **Max** | **Median** | **Min** | **Max** | **Median** |  |
| **Phylum** |  | | | | | | |
| Actinobacteria | 0.29 | 2.29 | 1.39 | 0.42 | 3.60 | 1.70 | 0.830 |
| Bacteroidetes | 7.59 | 45.93 | 31.15 | 1.02 | 31.08 | 18.00 | 0.830 |
| Firmicutes | 21.60 | 78.04 | 48.24 | 45.43 | 75.07 | 67.25 | 0.830 |
| Fusobacteria | 6.81 | 32.26 | 15.24 | 4.03 | 27.79 | 13.39 | 0.830 |
| Proteobacteria | 1.11 | 9.39 | 4.09 | 0.88 | 14.46 | 3.48 | 0.830 |
| **Class** |  | | | | | | |
| Actinobacteria | 0.01 | 0.13 | 0.05 | 0.01 | 0.25 | 0.07 | 0.942 |
| Coriobacteriia | 0.25 | 2.24 | 1.30 | 0.26 | 3.58 | 1.67 | 0.942 |
| Bacteroidia | 7.59 | 45.93 | 31.15 | 1.02 | 31.08 | 18.00 | 0.942 |
| Bacilli | 0.52 | 18.59 | 0.68 | 0.40 | 21.73 | 1.15 | 0.942 |
| Clostridia | 20.81 | 71.65 | 42.16 | 44.39 | 68.77 | 57.23 | 0.942 |
| Erysipelotrichi | 0.09 | 5.83 | 0.74 | 0.11 | 4.94 | 0.65 | 0.942 |
| Fusobacteriia | 6.81 | 32.26 | 15.24 | 4.03 | 27.79 | 13.39 | 0.942 |
| Alphaproteobacteria | 0.00 | 0.03 | 0.01 | 0.00 | 0.01 | 0.01 | 0.942 |
| Betaproteobacteria | 0.17 | 3.88 | 1.42 | 0.17 | 1.59 | 0.40 | 0.942 |
| Epsilonproteobacteria | 0.05 | 2.38 | 0.09 | 0.05 | 1.37 | 0.16 | 0.942 |
| Gammaproteobacteria | 0.40 | 5.46 | 1.42 | 0.30 | 13.96 | 1.57 | 0.942 |
| **Order** |  | | | | | | |
| Actinomycetales | 0.00 | 0.02 | 0.01 | 0.00 | 0.03 | 0.01 | 0.937 |
| Bifidobacteriales | 0.00 | 0.11 | 0.04 | 0.01 | 0.22 | 0.05 | 0.937 |
| Coriobacteriales | 0.25 | 2.24 | 1.30 | 0.26 | 3.58 | 1.67 | 0.937 |
| Bacteroidales | 7.59 | 45.93 | 31.15 | 1.02 | 31.08 | 18.00 | 0.937 |
| Bacillales | 0.00 | 0.08 | 0.02 | 0.01 | 0.08 | 0.03 | 0.937 |
| Lactobacillales | 0.39 | 9.89 | 0.57 | 0.31 | 21.47 | 0.96 | 0.937 |
| Turicibacterales | 0.00 | 15.41 | 0.12 | 0.02 | 4.23 | 0.16 | 0.937 |
| Clostridiales | 20.81 | 71.65 | 42.16 | 44.39 | 68.77 | 57.23 | 0.937 |
| Erysipelotrichales | 0.09 | 5.83 | 0.74 | 0.11 | 4.94 | 0.65 | 0.937 |
| Fusobacteriales | 6.81 | 32.26 | 15.24 | 4.03 | 27.79 | 13.39 | 0.937 |
| Burkholderiales | 0.17 | 3.88 | 1.42 | 0.17 | 1.59 | 0.40 | 0.937 |
| Campylobacterales | 0.05 | 2.38 | 0.09 | 0.05 | 1.37 | 0.16 | 0.937 |
| Aeromonadales | 0.02 | 1.32 | 0.04 | 0.02 | 2.10 | 0.05 | 0.937 |
| Enterobacteriales | 0.30 | 5.42 | 0.53 | 0.25 | 13.87 | 0.64 | 0.937 |
| **Family** |  | | | | | | |
| Actinomycetaceae | 0.00 | 0.02 | 0.00 | 0.00 | 0.02 | 0.01 | 0.981 |
| Bifidobacteriaceae | 0.00 | 0.11 | 0.04 | 0.01 | 0.22 | 0.05 | 0.981 |
| Coriobacteriaceae | 0.25 | 2.24 | 1.30 | 0.26 | 3.58 | 1.67 | 0.981 |
| Bacteroidaceae | 5.56 | 30.17 | 29.21 | 0.83 | 28.00 | 16.15 | 0.876 |
| Porphyromonadaceae | 0.00 | 0.03 | 0.02 | 0.00 | 3.42 | 0.01 | 0.981 |
| Prevotellaceae | 0.06 | 8.09 | 0.46 | 0.03 | 2.84 | 0.10 | 0.876 |
| [Paraprevotellaceae] | 0.02 | 9.65 | 2.01 | 0.01 | 2.93 | 0.13 | 0.876 |
| Bacillaceae | 0.00 | 0.04 | 0.01 | 0.01 | 0.05 | 0.01 | 0.876 |
| Paenibacillaceae | 0.00 | 0.02 | 0.01 | 0.00 | 0.03 | 0.00 | 0.981 |
| Planococcaceae | 0.00 | 0.03 | 0.00 | 0.00 | 0.03 | 0.01 | 0.981 |
| Staphylococcaceae | 0.00 | 0.01 | 0.00 | 0.00 | 0.03 | 0.01 | 0.981 |
| Enterococcaceae | 0.12 | 9.01 | 0.15 | 0.09 | 0.81 | 0.17 | 0.981 |
| Lactobacillaceae | 0.00 | 0.28 | 0.10 | 0.00 | 19.58 | 0.11 | 0.981 |
| Leuconostocaceae | 0.00 | 0.06 | 0.01 | 0.00 | 0.53 | 0.00 | 0.981 |
| Streptococcaceae | 0.23 | 0.41 | 0.34 | 0.22 | 1.16 | 0.37 | 0.981 |
| Turicibacteraceae | 0.00 | 15.41 | 0.12 | 0.02 | 4.23 | 0.16 | 0.981 |
| Clostridiales; f_other | 0.01 | 0.08 | 0.07 | 0.05 | 0.88 | 0.09 | 0.876 |
| Clostridiales; f_unclassified | 0.04 | 2.71 | 0.53 | 0.07 | 1.29 | 0.47 | 0.981 |
| Clostridiaceae | 1.92 | 26.53 | 10.05 | 5.44 | 30.17 | 19.28 | 0.981 |
| Lachnospiraceae | 2.17 | 44.33 | 15.00 | 18.95 | 38.45 | 26.10 | 0.876 |
| Peptococcaceae | 0.00 | 0.01 | 0.01 | 0.00 | 0.74 | 0.02 | 0.876 |
| Peptostreptococcaceae | 0.04 | 0.79 | 0.14 | 0.06 | 11.66 | 0.18 | 0.981 |
| Ruminococcaceae | 0.19 | 11.69 | 1.56 | 0.20 | 10.61 | 1.47 | 0.981 |
| Veillonellaceae | 0.82 | 16.16 | 6.81 | 0.42 | 18.45 | 2.85 | 0.876 |
| [Mogibacteriaceae] | 0.00 | 0.05 | 0.00 | 0.00 | 0.02 | 0.01 | 0.876 |
| Erysipelotrichaceae | 0.09 | 5.83 | 0.74 | 0.11 | 4.94 | 0.65 | 0.981 |
| Fusobacteriaceae | 6.81 | 32.26 | 15.24 | 4.03 | 27.79 | 13.39 | 0.981 |
| Alcaligenaceae | 0.17 | 3.88 | 1.42 | 0.17 | 1.59 | 0.40 | 0.876 |
| Campylobacteraceae | 0.00 | 0.45 | 0.02 | 0.00 | 0.92 | 0.00 | 0.981 |
| Helicobacteraceae | 0.03 | 2.26 | 0.09 | 0.04 | 0.92 | 0.16 | 0.981 |
| Succinivibrionaceae | 0.02 | 1.32 | 0.04 | 0.02 | 2.10 | 0.05 | 0.981 |
| Enterobacteriaceae | 0.30 | 5.42 | 0.53 | 0.25 | 13.87 | 0.64 | 0.981 |
| **Genus** |  | | | | | | |
| Actinomyces | 0.00 | 0.02 | 0.00 | 0.00 | 0.02 | 0.01 | 0.985 |
| Bifidobacterium | 0.00 | 0.10 | 0.04 | 0.01 | 0.22 | 0.05 | 0.985 |
| Collinsella | 0.25 | 1.98 | 1.29 | 0.22 | 3.58 | 1.60 | 0.985 |
| Slackia | 0.00 | 0.26 | 0.01 | 0.00 | 0.12 | 0.00 | 0.985 |
| Bacteroides | 5.56 | 30.17 | 29.21 | 0.83 | 28.00 | 16.15 | 0.985 |
| Parabacteroides | 0.00 | 0.03 | 0.02 | 0.00 | 3.42 | 0.01 | 0.985 |
| Prevotella | 0.06 | 8.09 | 0.46 | 0.03 | 2.84 | 0.10 | 0.985 |
| [Prevotella] | 0.02 | 8.53 | 2.01 | 0.01 | 2.93 | 0.13 | 0.985 |
| Bacillus | 0.00 | 0.03 | 0.01 | 0.01 | 0.04 | 0.01 | 0.985 |
| Paenibacillus | 0.00 | 0.02 | 0.01 | 0.00 | 0.03 | 0.00 | 0.985 |
| Enterococcaceae; g_other | 0.00 | 0.28 | 0.01 | 0.00 | 0.05 | 0.01 | 0.985 |
| Enterococcus | 0.11 | 8.70 | 0.15 | 0.08 | 0.76 | 0.16 | 0.985 |
| Lactobacillus | 0.00 | 0.28 | 0.10 | 0.00 | 19.58 | 0.11 | 0.985 |
| Leuconostocaceae; g_unclassified | 0.00 | 0.06 | 0.01 | 0.00 | 0.51 | 0.00 | 0.985 |
| Streptococcus | 0.23 | 0.41 | 0.32 | 0.22 | 1.16 | 0.35 | 0.985 |
| Turicibacter | 0.00 | 15.41 | 0.12 | 0.02 | 4.23 | 0.16 | 0.985 |
| Clostridiales; f_other; g_other | 0.01 | 0.08 | 0.07 | 0.05 | 0.88 | 0.09 | 0.985 |
| Clostridiales; f_unclassified; g_unclassified | 0.04 | 2.71 | 0.53 | 0.07 | 1.29 | 0.47 | 0.985 |
| Clostridiaceae; g_other | 1.14 | 12.85 | 5.73 | 2.78 | 14.85 | 10.74 | 0.985 |
| Clostridiaceae; g_unclassified | 0.68 | 8.11 | 3.64 | 2.17 | 14.49 | 6.29 | 0.985 |
| Clostridium | 0.06 | 1.09 | 0.09 | 0.07 | 4.36 | 0.26 | 0.985 |
| SMB53 | 0.01 | 0.16 | 0.07 | 0.03 | 0.29 | 0.09 | 0.985 |
| Sarcina | 0.00 | 8.28 | 0.01 | 0.01 | 0.99 | 0.02 | 0.985 |
| Lachnospiraceae; g_other | 0.02 | 1.09 | 0.28 | 0.12 | 1.13 | 0.40 | 0.985 |
| Lachnospiraceae; g_unclassified | 0.64 | 17.20 | 1.83 | 0.49 | 7.38 | 2.16 | 0.985 |
| Blautia | 0.57 | 18.44 | 7.65 | 4.17 | 12.95 | 10.03 | 0.985 |
| Coprococcus | 0.00 | 0.57 | 0.12 | 0.08 | 0.41 | 0.22 | 0.985 |
| Dorea | 0.17 | 6.56 | 2.06 | 1.52 | 4.48 | 3.00 | 0.985 |
| Epulopiscium | 0.00 | 0.02 | 0.01 | 0.00 | 0.21 | 0.01 | 0.985 |
| Roseburia | 0.00 | 0.34 | 0.01 | 0.00 | 0.37 | 0.02 | 0.985 |
| [Ruminococcus] | 0.77 | 8.70 | 2.86 | 3.30 | 15.72 | 11.79 | 0.510 |
| Peptococcus | 0.00 | 0.01 | 0.01 | 0.00 | 0.74 | 0.02 | 0.985 |
| Peptostreptococcaceae; g_unclassified | 0.04 | 0.79 | 0.14 | 0.06 | 11.66 | 0.18 | 0.985 |
| Ruminococcaceae; g_unclassified | 0.17 | 10.48 | 1.46 | 0.15 | 9.23 | 1.23 | 0.985 |
| Faecalibacterium | 0.01 | 1.18 | 0.10 | 0.01 | 1.03 | 0.14 | 0.985 |
| Oscillospira | 0.00 | 0.20 | 0.01 | 0.00 | 0.32 | 0.01 | 0.985 |
| Ruminococcus | 0.00 | 0.12 | 0.01 | 0.00 | 0.03 | 0.02 | 0.985 |
| Veillonellaceae; g_other | 0.00 | 0.08 | 0.01 | 0.00 | 0.19 | 0.01 | 0.985 |
| Dialister | 0.00 | 0.04 | 0.00 | 0.00 | 0.62 | 0.00 | 0.985 |
| Megamonas | 0.61 | 16.07 | 6.56 | 0.38 | 18.40 | 1.49 | 0.985 |
| Phascolarctobacterium | 0.04 | 1.60 | 0.29 | 0.03 | 1.06 | 0.13 | 0.985 |
| [Mogibacteriaceae]; g_unclassified | 0.00 | 0.05 | 0.00 | 0.00 | 0.02 | 0.01 | 0.985 |
| Erysipelotrichaceae; g_unclassified | 0.01 | 2.88 | 0.20 | 0.02 | 3.86 | 0.07 | 0.985 |
| Allobaculum | 0.00 | 0.33 | 0.04 | 0.00 | 0.85 | 0.10 | 0.985 |
| Catenibacterium | 0.01 | 1.10 | 0.21 | 0.02 | 0.95 | 0.08 | 0.985 |
| Coprobacillus | 0.00 | 0.01 | 0.00 | 0.00 | 0.01 | 0.01 | 0.985 |
| [Eubacterium] | 0.03 | 1.82 | 0.15 | 0.06 | 1.02 | 0.20 | 0.985 |
| Fusobacteriaceae; g_other | 0.39 | 1.42 | 0.76 | 0.40 | 1.77 | 0.88 | 0.985 |
| Cetobacterium | 0.00 | 0.06 | 0.01 | 0.00 | 0.07 | 0.01 | 0.985 |
| Fusobacterium | 5.78 | 31.41 | 14.42 | 3.51 | 25.94 | 12.29 | 0.985 |
| Sutterella | 0.17 | 3.88 | 1.42 | 0.17 | 1.59 | 0.40 | 0.985 |
| Campylobacter | 0.00 | 0.45 | 0.02 | 0.00 | 0.92 | 0.00 | 0.985 |
| Helicobacter | 0.03 | 2.24 | 0.09 | 0.04 | 0.92 | 0.16 | 0.985 |
| Succinivibrionaceae; g_unclassified | 0.00 | 0.85 | 0.01 | 0.00 | 0.06 | 0.00 | 0.985 |
| Anaerobiospirillum | 0.02 | 1.16 | 0.04 | 0.01 | 2.07 | 0.04 | 0.985 |
| Enterobacteriaceae; g_other | 0.01 | 0.02 | 0.01 | 0.00 | 0.04 | 0.01 | 0.985 |
| Enterobacteriaceae; g_unclassified | 0.28 | 5.38 | 0.51 | 0.24 | 13.83 | 0.60 | 0.985 |
| Proteus | 0.00 | 0.03 | 0.02 | 0.00 | 0.03 | 0.02 | 0.985 |

**Supplementary table 3.** Bacterial taxa detected in fecal samples from healthy fed a hydrolyzed protein diet for 6 weeks. * p-values have been adjusted for multiple comparisons by the Benjamin & Hochberg FDR.
